# Physical, Chemical, and Sensory Properties of a Turmeric-Fortified Pineapple Juice Beverage

**DOI:** 10.3390/foods12122323

**Published:** 2023-06-09

**Authors:** Xiuxiu Sun, Peter A. Follett, Marisa M. Wall, Keegan S. Duff, Xiaohua Wu, Chang Shu, Anne Plotto, Peishih Liang, Dara G. Stockton

**Affiliations:** 1 United States Department of Agriculture, Agricultural Research Service, Daniel K. Inouye U.S. Pacific Basin Agricultural Research Center, 64 Nowelo Street, Hilo, HI 96720, USA; 2 United States Department of Agriculture, Agricultural Research Service, U.S. Horticultural Research Laboratory, 2001 South Rock Road, Fort Pierce, FL 34945, USA

**Keywords:** *Curcuma longa*, antioxidant, phenolic compound, volatiles, turmerone, sensory

## Abstract

Beverage mixtures based on pineapple (*Ananas comosus*) and turmeric (*Curcuma longa*) juice as a ready-to-drink product were developed, and their physicochemical, nutritional, and sensory properties were evaluated. Four different concentrations of turmeric juice (5%, 10%, 15%, and 20% (*v*/*v*)) were added to pineapple juice to make turmeric-fortified pineapple (TFP) juice samples. Pineapple juice without turmeric was the control. The L*, a*, b*, titratable acidity (TA), total antioxidant capacity, and %DPPH scavenging values, as well as the concentrations of the phenolic compounds curcumin and demethoxycurcumin, were significantly increased with increasing turmeric concentration. Thirty volatile compounds were detected in the mixed juice samples with turmeric. Most of the turmeric-specific compounds, including monoterpenes, sesquiterpenes and turmerones, were detected in the TFP juice samples. While the antioxidant activity of the juice samples increased with increasing turmeric concentration, the pineapple juice fortified with 10% turmeric (10%T) had the best overall quality as determined by panelists. Greater concentrations of turmeric were associated with decreased palatability due to reduced mouthfeel and sweetness and increased aftertaste and sourness. These results suggest that the 10%T juice could be developed into a commercial functional beverage with increased overall flavor and nutritional quality.

## 1. Introduction

Turmeric, *Curcuma longa*, is a perennial plant in the ginger family, known for its aromatic, bright yellow rhizomes and used widely in Asian cooking and traditional medicine practices [1]. Native to India and South Asia, it has been cultivated in these regions for centuries, and it is also cultivated on a small scale in tropical regions of the Americas and the Pacific basin, including Hawaii. The production of turmeric was 515,000 pounds in Hawaii in 2021, valued at USD 2.77 million. It was the top crop by value for 2021 [2]. Turmeric plants require temperatures between 20 °C and 35 °C and ample rainfall [3]. Plants can grow to a height of 1 m, with simple oblong or elliptical leaves. Turmeric powder, a yellow-brown spice which is the primary source of economic value for the plant, is produced via the pulverization of the plant’s rough, segmented rhizome [1]. In recent years, the demand for and consumption of turmeric and ginger have increased, and the US has become a top importer of these products from China and India [4].

Turmeric is a rich source of most essential amino acids (6–8% of dry weight (DW)), dietary fiber and minerals (5–14% DW), and many phytochemicals including diarylheptanoids (1–6% DW), and the plant is reported to possess numerous health benefits including antidiabetic, antimicrobial, antioxidant, and anti-inflammatory properties [1,5,6], thus leading it to be dubbed a popular “superfood” in American marketing programs. Curcumin, or diferuloylmethane, is the primary phenolic compound in turmeric rhizomes, and has been studied intensively in cancer research, including its applications in radiotherapy and chemotherapy to reduce adverse side effects, as well as its effects in cancer development and proliferation [7,8]. It appears to function by modulating cell signaling pathways [9] and is found in several biochemical forms within the rhizome, as curcuminoids (curcumin (77%), demethoxycurcumin (17%), and bis-demethoxycurcumin (3%), each with varying activity and targets [10]. In complex, curcumin is typically taken orally via nutritional supplementation, and 50–100 mg is estimated to be consumable via regular dietary sources with no adverse effects [11,12]. In the United States, turmeric is Generally Recognized as Safe (GRAS), and curcumin has an approved consumption level of 1–3 mg/kg body weight [10]. Several studies evaluating potential toxicity or adverse effects have found little to no risks even at doses as high as 12,000 mg daily [13].

Due to its high nutritional and medicinal value, turmeric has been increasingly used in popular markets to produce functional drinks such as turmeric tea and turmeric golden root juice. However, turmeric drinks have a strong earthy and spicy taste profile and are not always perceived well by consumers, which greatly limits their marketability at higher concentrations. Pineapple (*Ananas comosus*), on the other hand, is one of the most popular tropical fruit crops in Hawaii with high yield (170,000 tons in 2020) [14]. Pineapple juice is preferred by consumers for its unique aroma and flavor, which come from a set of amino acids, amines, phenolic compounds, and furanone [15]. Pineapple juice contains 16% carbohydrates, 84% water, crude fiber, protein, and other micronutrients such as calcium (1% of the daily value (DV)), potassium (3% DV), and manganese (53% DV) that are essential for maintaining balanced nutrition [16]. It can be recommended to people suffering from certain disorders as part of a medically prescribed diet [17]. Bromelain, the digestive enzyme in pineapple, has pain-relieving and anti-inflammatory properties [14]. As consumer demand for natural “superfood” beverages and clean label foods increases [18,19], ready-to-serve products can be prepared by mixing two or more fruit juices in different proportions resulting in improved aroma, taste and nutritional content [20]. In this research, Hawaiian organic turmeric and pineapple were used for the development of a drink product. The objectives of this study were to develop a turmeric-fortified functional drink based on pineapple juice, and to evaluate its physical, chemical, sensory, and functional properties. The positive outcomes of this study provide greater market potential for turmeric and expand the health benefit of pineapple juice. Our results will support local farms and benefit the local economy.

## 2. Materials and Methods

### 2.1. Materials and Juice Preparation

Turmeric juice was provided by Crown Pacific International (Hilo, HI, USA). Pineapple fruit puree was obtained from Maui Fruit Jewels (Wailuku, HI, USA). Turmeric-fortified pineapple (TFP) drinks were developed by mixing 5–20% (*v*/*v*) of turmeric juice with 80–95% (*v*/*v*) pineapple juice. The juice samples were pasteurized at 71 °C for 30 s. There were five juice samples, including four juice samples with turmeric (5%T, 10%T, 15%T and 20%T, representing adding 5%, 10%, 15% and 20% turmeric juice into pineapple juice, respectively) and pineapple juice without turmeric as a control.

### 2.2. Color and pH

Color and pH were measured using a colorimeter and pH meter, respectively. Drink color was measured at three points using a Minolta Chroma Meter (model CR-300, Minolta Corp., Ramsey, NJ, USA) and recorded as CIE L*, a*, and b*, where L* indicates lightness read from 0 (black) to 100 (white). A positive a* value indicates red color while a negative a* value represents green color. Similarly, positive and negative b* values indicate yellow and blue colors, respectively [21]. ∆E* = [(∆L*)^2^ + (∆a*)^2^ + (∆b*)^2^]^1/2^. The final value indicates the total change of lightness, red/greenness, and yellow/blueness.

### 2.3. Total Soluble Solids (TSS) and Titratable Acidity (TA)

The total soluble solids (TSS) content was determined using a digital refractometer (PAL-3, ATAGO U.S.A., Inc., Bellevue, WA, USA), and reported as °Brix. The titratable acidity (TA) was determined using an acidity meter (GMK-835F, ATAGO^®^, ATAGO U.S.A., Inc., Bellevue, WA, USA), and expressed as %TA [22].

### 2.4. Volatiles

Volatiles were determined by gas chromatography-mass spectrometry (GC-MS) using the method described in previous research, with modifications [23]. Six milliliters of juice were transferred to 20-mL vials and the vials were crimp-capped with Teflon/silicone septa. Juice samples were analyzed by GC-MS (Model 8890, Agilent, Santa Clara, CA, USA) equipped with a 19091S-433 HP-5ms 5% Phe column (30 m × 0.25 mm i.d., 0.25 µm film thickness, Agilent, Santa Clara, CA, USA). The headspace conditions were: oven temperature 120 °C, loop 120 °C, transfer line 130 °C, vial equilibrium 10 min, injection duration 0.5 min, and cycle time 45 min. The GC conditions (35 min run) were: inlet 220 °C, flow rate 24.2 mL/min, pressure 3 mL/min, split ratio 20:1, gas saver on 3.0 mL/min after 3 min, oven 40 °C with a 2 min hold, and 10 °C/min ramp to 280 °C with a 9 min hold. The MS conditions were: transfer line 290 °C, MS-Q 150 °C, MS 250 °C, EI voltage 70 eV, and scanning range 45 to 350 *m*/*z*. Volatile compounds were identified by comparison of their mass spectra with NIST library entries (>90% match) [23]. Eucalyptol, *γ*-terpinene, terpinolene and *α*-turmerone standards were used to confirm those compounds.

### 2.5. Total Antioxidant Capacity

The total antioxidant capacity of the juice samples was determined with an antioxidant assay kit (MAK334, Sigma-Aldrich, St. Louis, MO, USA) according to the manufacturer’s recommendations. Cu^2+^ is reduced by antioxidants to Cu^+^, which specifically forms a colored complex with a dye reagent. The color intensity at 570 nm is proportional to the total antioxidative capacity of the sample. Juice samples were centrifuged at 10,000× *g* for 15 min at room temperature. Supernatants were diluted with distilled water (1:1) and 20 µL of diluted sample was mixed with 100 µL reaction mix in a clear 96-well plate. Following 10 min of incubation, the absorbance was measured at 570 nm using a SpectraMax M2 microplate reader (Molecular Devices, LLC, San Jose, CA, USA). Absorbance values were compared to a calibration curve prepared with 300–1000 µM trolox solutions.

### 2.6. DPPH (1,1-Diphenyl-2-Picryl-Hydrazyl) Assay

The DPPH (1,1-diphenyl-2-picryl-hydrazyl) assay was carried out using a scavenging assay that detects juice sample DPPH radical inhibition [24]. The working DPPH solution was obtained by dissolving DPPH powder in methanol to obtain a concentration of 0.2 mM. For each sample, 100 µL was diluted with 900 µL of water, 100 µL of samples or a blank (distilled water) were placed in individual wells of a 96-well polystyrene microplate, and 100 µL of working DPPH solution was then added. As the sample background, 100 µL of samples were added in the plate with the addition of 100 µL of methanol. After 30 min reaction at room temperature, the absorbance was measured at 515 nm using a SpectraMax M2 microplate reader (Molecular Devices, LLC, San Jose, CA, USA). The percentage of radical scavenging was obtained using the following expression:

$$\mathrm{DPPH}\;\mathrm{inhibition}\;\mathrm{percentage}\;=\frac{\left(\mathrm{Abs}\;\mathrm{Sample}+\mathrm{DPPH}\right)-\left(\mathrm{Abs}\;\mathrm{Blank}\right)}{\left(\mathrm{Abs}\;\mathrm{DPPH}\right)-\left(\mathrm{Abs}\;\mathrm{Solvent}\right)}\times 100$$

### 2.7. Phenolic Compounds

Aliquots (4 mL) of turmeric/pineapple juice mixture were transferred into 5 mL vials and then submerged in liquid nitrogen and freeze-dried using a Freezone6 Labconco unit (Kansas City, MO, USA). Ending conditions for the freeze dryer were 51 °C cooling coil and 0.021 mBar vacuum. The freeze-dried samples were weighed, and a stainless-steel grinder ball was added. The samples were then submerged in liquid nitrogen and cryogenically milled using a grinder (Spex SamplePrep 2010; Metuchen, NJ, USA) for 30 s at 1750 rpm. After milling, samples were reconstituted with 4 mL of methanol. Then 200 µL of each sample was transferred to 2 mL centrifuge tubes and diluted with 1.6 mL methanol, vortexed, and centrifuged for 5 min at 4 °C at 18,213 rpm. The supernatant was transferred to 250 µL insert vials and 1 µL injection volumes were used for analysis. Samples were stored at 6 °C prior to analysis.

Two phenolic compounds, curcumin and demethoxycurcumin, were quantified using liquid chromatography-quadrupole time-of-flight mass spectrometry (LC-QTOF-MS) according to a modification of the method of Yang et al. [25]. The analysis was performed using an Agilent 1290 Infinity II LC system with a high-speed pump, autosampler, and dual source AJS with a G6545B quadrupole TOF-MS (Agilent technologies, Santa Clara, CA, USA). Separation was achieved with a Zorbax Extend-C18 (4.6 mm I.D. × 150 mm length; 5-µm) column and a matching Zorbax Extend-C18 analytical guard column (4.6 mm I.D. × 12.5 mm length; 5-µm) (Agilent Technologies; Palo Alto, CA, USA). An isocratic flow of 0.6 µL/min mobile phase consisting of 70% acetonitrile and 29.9% water with 0.1% ethyl alcohol at 30 °C was used, with a 15 min run time followed by a 5 min post wash cycle. The injection volume was 1 µL. Settings for the dual source AJS system were: negative mode, gas temperature of 275 °C, drying gas 10 L/min, nebulizer 50 psi, sheath gas 325 °C, sheath gas 12 L/min, Vcap 3500 V, and nozzle voltage 500 V. The TOF was tuned, and transmission was tuned for optimal performance from 50 to 1700 *m*/*z*. Masshunter WorkStation Quantitative Analysis software (Version 10.1; Agilent Technologies; Palo Alto, CA, USA) was employed for data analysis. Analytes were confirmed by matching chromatography retention time and accurate mass data to known standards. Additional confirmation was obtained by confirming fragmentation patterns of samples to those of known standards [25]. The concentrations were calculated using standard curves obtained by diluting standards (Sigma-Aldrich, St. Louis, MO, USA) in methanol.

### 2.8. Sensory Evaluation

TFP juice samples from the four treatments (5%T, 10%T, 15%T and 20%T) and a 100% pineapple juice (control) were evaluated by 10 panelists and used to evaluate tropical fruit products. Panelists were asked about flavor, sweetness, sourness, mouthfeel (rough to smooth), aftertaste, and overall quality using a 0–10 intensity scale (0 = none, 10 = high intensity). Reference standards for sweetness and sourness (intensity 1, 5, and 10) were provided at the following concentrations: 1%, 5%, and 10% (*w*/*v*) of sucrose for intensities of 1, 5, and 10, respectively, and 0.025%, 0.1%, and 0.2% (*w*/*v*) of citric acid for intensities of 1, 5, and 10, respectively. Two training sessions were conducted with panelists to discuss descriptors: flavor, mouthfeel, aftertaste, and overall quality. Samples, three replications in separate sessions, were presented as 20 mL in 50-mL plastic cups with lids (SOLO, Urbana, IL, USA) at 20 ± 2 °C.

### 2.9. Statistical Analysis

Data were organized and graphed using Excel (Microsoft Corp., Seattle, WA, USA) and analyzed using JMP statistical analysis software (version 16; SAS Institute, Cary, NC, USA). Analysis of variance (ANOVA) was used to evaluate the effect of treatments on juice quality attributes. For significant treatment effects, means separation was performed using a Tukey’s HSD test at α = 0.05. At least three replications were conducted for all experiments.

Correlation analysis was performed among phytochemical parameters and sensory properties and among phenolic compounds and antioxidant capacities using the JMP statistical analysis software. The correlation coefficients were analyzed with the Pearson method at α = 0.05. A level of *p* < 0.05 or less was considered to be significant.

## 3. Results

### 3.1. Physicochemical Properties

**Figure 1:**
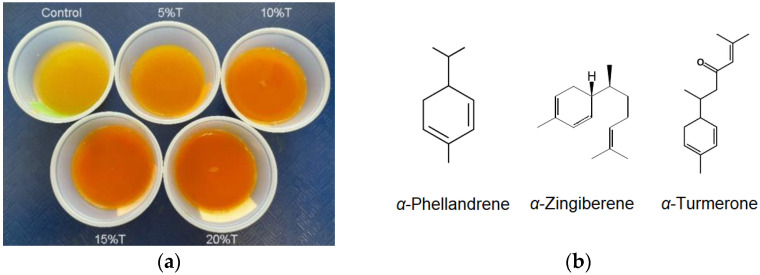
Turmeric-fortified pineapple (TFP) juices (**a**) and the chemical structures of the main volatile compounds (**b**) (control: 100% pineapple juice; 5%T: 95% pineapple juice + 5% turmeric juice; 10%T: 90% pineapple juice + 10% turmeric juice; 15%T: 85% pineapple juice + 15% turmeric juice; and 20%T: 80% pineapple juice + 20% turmeric juice).

The physicochemical properties of TFP juices including color, pH, total soluble solids (TSS) and titratable acidity (TA) are shown in Table 1. The effect of turmeric on juice color was significant, with L*, a*, b*, and ∆*E* values increasing with increasing turmeric concentration (Table 1). The 10%T, 15%T, and 20%T samples showed dark orange color (Figure 1a). The positive a* values for the 10%T (1.74), 15%T (3.97) and 20%T (5.41) samples indicate that the color of these samples was reddish. The a* and b* values increased with the concentration of turmeric, indicating stronger degrees of red and yellow color [26]. A similar trend was noticed in a curcuma-based herbal drink [27]. The pH and TA of juice samples were 3.64–3.90, and 0.52–0.67% respectively (Table 1), and their values increased with an increasing concentration of turmeric juice (Table 1). Ogori et al. [28] reported a similar pH trend in ginger, pineapple, and turmeric juice mixes. Guerra et al. [26], however, observed no change in pH and TA values with increasing concentrations of turmeric juice in yogurt. In our study, the pH of all the juice samples was below 4, which is acidic enough to deter most types of microbial growth [22]. The 100% pineapple juice showed the highest TSS with 13.10 °Brix, and 20%T had the lowest TSS with 11.70 °Brix (Table 1). The decreases in TSS are probably due to the lower sugar content in the turmeric component of the juice mixture relative to 100% pineapple juice [28].

### 3.2. Volatiles

**Figure 2:**
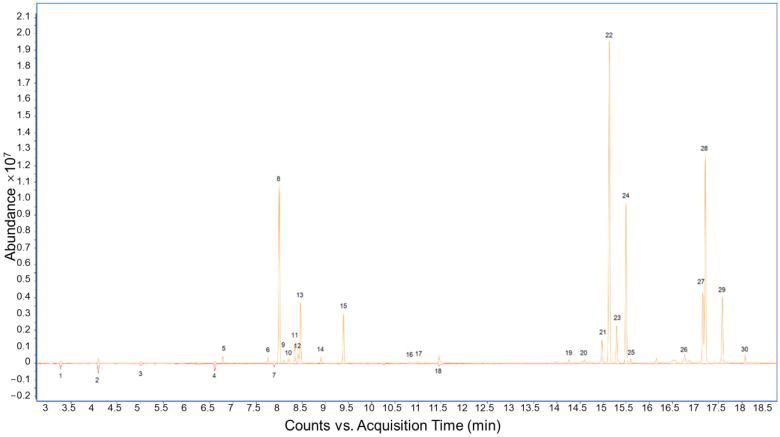
Gas chromatography-mass spectrometry (GC-MS) chromatograms of 80% pineapple juice + 20% turmeric juice (20%T; top) and 100% pineapple juice (control; bottom).

The detected volatile compounds in the juice samples are listed in Table 2 and Figure 2. Thirty compounds were detected in the mixed juice samples with turmeric, with only six of those compounds (methyl butyrate, methyl 2-methylbutyrate, furfural, methyl hexanoate, ethyl hexanoate, and 5-hydroxymethylfurfural) detected in 100% pineapple juice (Table 2 and Figure 2). The turmeric-specific compounds included monoterpenes, sesquiterpenes and the three turmerone compounds (#27–29; Table 2). Those compounds typically have woody, earthy, or medicinal characteristics, and might contribute to a medicinal and earthy odor perceived in the turmeric juice [29]. They all increased with increasing turmeric concentration in the juice mixture. Previous research showed that the most abundant compounds in turmeric essential oils are *ar*-turmerone, followed by *α*-turmerone, *β*-turmerone, terpinolene, *β*-sesquiphellandrene, *α*-zingiberene, *β*-caryophyllene, *ar*-curcumene, and eucalyptol [30,31]. In addition, (*Z*)-*β*-farnesene, *β*-bisabolene, *α*-phellandrene, and terpinolene have also been detected in turmeric juice [31]. Curcumene, *ar*-turmerone and *β*-sesquiphellandrene were confirmed as the main pharmacological volatiles of turmeric [32,33]. *a*-Phellandrene is a plant metabolite that is a colorless to light yellow oily liquid, with an odor similar to black pepper, that can act as an antimicrobial agent [30]. α-Zingiberene, a signature compound for plants in the Zingiberaceae family, is one of the main constituents of turmeric plants with anti-inflammatory properties [31]. Tumerones, including *a*-turmerone, have been reported to have anti-inflammatory, immunomodulatory, antifungal, and antiproliferative activities [31]. The chemical structures of α-phellandrene, α-zingiberene, and *α*-turmerone are shown in Figure 1b. In our research, all the main compounds were detected in the TFP juices (Table 2 and Figure 2).

### 3.3. Total Antioxidant Capacity and DPPH Inhibition

The total antioxidant capacity and the DPPH inhibition percentage are shown in Table 3. The total antioxidant capacity of TFP juice samples was around 1000 Trolox (μM), which was significantly higher than the control (Table 3). The DPPH inhibition percentage of TFP samples was up to 90%; however, the DPPH inhibition percentage for the control was 71%, which was significantly lower than the TFP juices (Table 3). Overall, antioxidant activity increased with increased turmeric concentration. Idowu-Adebayo et al. [34] observed a similar trend in a turmeric-fortified milk product. Both phenolic and flavonoid compounds in turmeric juice have been found to increase antioxidant activity [27], especially curcumin and demethoxycurcumin [35,36]. Tannin in turmeric can also show strong antioxidant activity [37]. While the TFP juices showed higher total antioxidant capacity, there was no significant difference among the different turmeric concentrations. TFP juices with 10% or higher turmeric showed significantly higher DPPH inhibition percentage than juice with 5% turmeric, and there was no significant difference among 10%T, 15%T, and 20%T (Table 3). Similarly, Idowu-Adebayo et al. [34] showed that adding either 2% or 6% turmeric into whole and skimmed milk did not significantly change the total antioxidant capacity.

### 3.4. Phenolic Compounds

**Figure 3:**
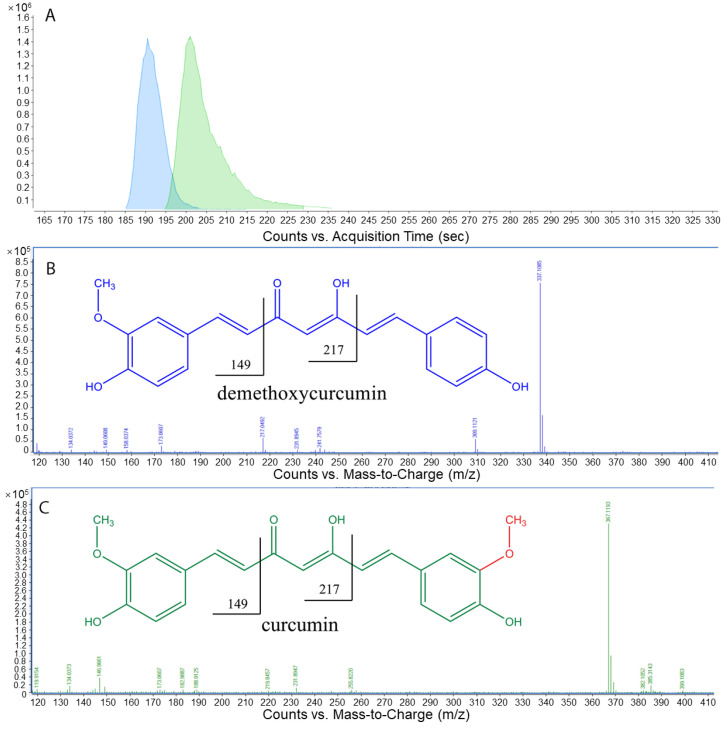
(**A**–**C**) Extracted ion chromatograms (EIC) and respective averaged background subtracted chromatograms of demethoxycurcumin and curcumin in turmeric-fortified pineapple (TFP) juice samples.

The two main phenolic compounds in turmeric, curcumin and demethoxycurcumin, were analyzed using LC-QTOF-MS (Figure 3). Curcumin and demethoxycurcumin are well-recognized components of turmeric known to contribute to the prevention of multiple inflammation-type disorders [38,39]. Recovery of standards and samples was evaluated as a function of the extraction solvent, including acetonitrile, absolute ethanol, and methanol. Methanol was selected as having the highest chromatographic resolution and recovery [38]. In the juice samples with turmeric, concentrations of curcumin ranged from 19.40 to 47.50 mg/L and demethoxycurcumin concentrations ranged from 2.38 to 11.85 mg/L. The concentrations of the two phenolic compounds increased significantly as turmeric content in juice blend increased (Table 4). Yang reported recovery of curcumin at 0.11 mg/kg, 14.4 mg/kg and 165.3 mg/kg for water, 50% ethanol and 95% ethanol, respectively, from the tubers of *Curcuma longa* [25]. The levels reported in our study for curcumin and demethoxycurcumin are higher than those reported by Yang et al. [25] and another report that used acetonitrile as an extraction and injection solvent for dried turmeric powder [40]. A positive correlation between phenolic compounds and antioxidant properties was found in the juice samples (Table 5). Higher concentrations of curcumin and demethoxycurcumin contributed to higher total antioxidant capacity and DPPH inhibition percentage [39]. During the method development in our study, freeze-drying was shown to improve recovery. This was attributed to moisture removal and reduction of matrix effects. These analytes can readily shift from keto to enol form. Formic acid was added to the mobile phase to promote the protonated keto form during chromatography and ionization [38].

### 3.5. Sensory Evaluation

**Figure 4:**
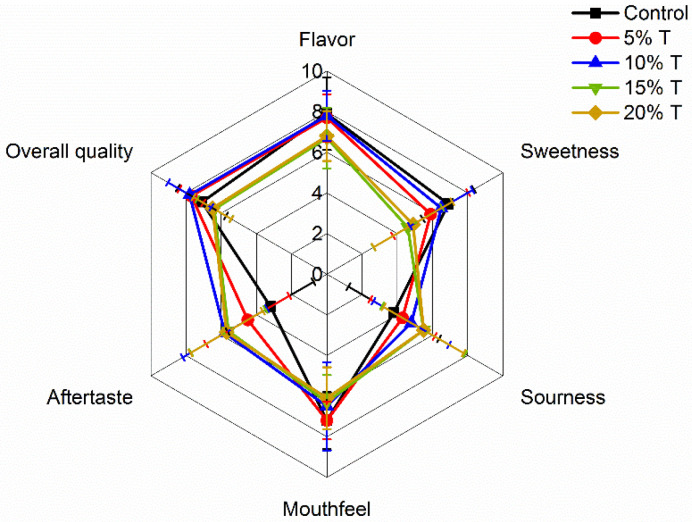
Sensory properties of turmeric-fortified pineapple (TFP) juice samples (control: 100% pineapple juice; 5%T: 95% pineapple juice + 5% turmeric juice; 10%T: 90% pineapple juice + 10% turmeric juice; 15%T: 85% pineapple juice + 15% turmeric juice; and 20%T: 80% pineapple juice + 20% turmeric juice).

The sensory profile of each formulation is illustrated in the spider web chart in Figure 4. The 10%T formulation showed almost all attributes scoring higher than 6, suggesting that the panelists judged this juice to be of higher quality than the others. Drink mixtures with 15% or 20% turmeric were rated as higher in aftertaste and sourness, but lower in flavor, sweetness, and mouthfeel, suggesting these formulations were overall less palatable. In a similar study, Ogori et al. [28] found that a juice mixture with 80% pineapple, 10% turmeric, and 10% ginger was rated higher than the juice with 20–40% turmeric, indicating that at a certain point increasing the turmeric and decreasing pineapple juice made the mixed juice less preferred. While in some cases higher turmeric drinks have previously been reported as preferred, this may be due to the amount of sugar that was added to the formulations, rather than the turmeric itself [27]. The Pearson correlation coefficients between sensory scorings, and between phytochemical properties and the main volatiles of the juice samples, are listed in Table 6. A significant (*p* < 0.01) negative correlation between sourness and TSS, and a significant (*p* < 0.05) positive correlation between sourness and TA, phenolic compounds and most volatiles, were noticed in the juices. An opposite trend was found for mouthfeel. A significant (*p* < 0.05) positive correlation between the main volatile compounds, including turmerones and α-zingiberene, and aftertaste was found in the juices (Table 6). Turmerones are the main bitter components of turmeric [41]. α-Zingiberene is a monocyclic sesquiterpene that has a bitter and spicy taste [42]. *α*-Phellandrene has black pepper odor character. Those compounds significantly (*p* < 0.05) affect the aftertaste of the juices. A comparable trend was found in a previous study [30].

## 4. Conclusions

Our study demonstrates the potential for the utilization of turmeric juice to fortify a tropical fruit beverage. TFP juice with 90:10 pineapple to turmeric (10%T, *v*/*v*) received the highest sensory score for most sensory attributes, while also possessing higher antioxidant activity with higher total antioxidant capacity and DPPH inhibition percentage. The TFP juice samples also showed significantly higher demethoxycurcumin and curcumin content and better physicochemical properties than 100% pineapple juice. The main volatile compounds, including *α*-turmerone, *α*-phellandrene, and *α*-zingiberene, were only detected in TFP juice samples. Our results show that pineapple juice can be improved with the addition of turmeric juice to produce a functional beverage with higher antioxidant and nutritional quality.

## Figures and Tables

**Table 1:** The physicochemical properties (color, pH, total soluble solids (TSS) and titratable acidity (TA)) of turmeric-fortified pineapple (TFP) juices (control: 100% pineapple juice; 5%T: 95% pineapple juice + 5% turmeric juice; 10%T: 90% pineapple juice + 10% turmeric juice; 15%T: 85% pineapple juice + 15% turmeric juice; and 20%T: 80% pineapple juice + 20% turmeric juice).

Samples	Color				pH	TSS (°Brix)	TA (%)
	L*	a*	b*	∆*E*			
Control	27.29 ± 0.79 e	−1.73 ± 0.34 e	4.59 ± 0.49 e		3.64 ± 0.02 d	13.10 ± 0.02 a	0.52 ± 0.02 d
5%T	31.86 ± 0.16 d	−0.70 ± 0.05 d	11.59 ± 0.22 d	8.43 ± 0.24 d	3.77 ± 0.01 c	12.67 ± 0.06 ab	0.56 ± 0.01 cd
10%T	33.54 ± 0.31 c	1.74 ± 0.06 c	14.36 ± 0.16 c	12.11 ± 0.26 c	3.79 ± 0.01 bc	12.03 ± 0.64 bc	0.59 ± 0.01 bc
15%T	34.78 ± 0.10 b	3.97 ± 0.05 b	15.56 ± 0.10 b	14.46 ± 0.09 b	3.81 ± 0.01 b	11.80 ± 0.35 bc	0.61 ± 0.02 b
20%T	36.29 ± 0.52 a	5.41 ± 0.10 a	16.56 ± 0.12 a	16.61 ± 0.25 a	3.90 ± 0.01 a	11.70 ± 0.02 c	0.67 ± 0.02 a

Mean values ± standard deviations followed by different letters within a column indicate significant differences using a Tukey’s HSD test at α = 0.05.

**Table 2:** Volatile compounds in turmeric-fortified pineapple (TFP) juice samples (control: 100% pineapple juice; 5%T: 95% pineapple juice + 5% turmeric juice; 10%T: 90% pineapple juice + 10% turmeric juice; 15%T: 85% pineapple juice + 15% turmeric juice; and 20%T: 80% pineapple juice + 20% turmeric juice).

			Average Abundance (Total Ion Current × 10^6^)				
Pk#	RT	Compound Name	Control	5%T	10%T	15%T	20%T
1	3.28	Methyl butyrate	2.99 ± 0.17 ab	3.52 ± 0.43 a	3.53 ± 0.28 a	2.57 ± 0.17 b	2.58 ± 0.19 b
2	4.09	Methyl 2-methylbutyrate	7.22 ± 0.40 b	8.69 ± 0.13 ab	7.44 ± 0.20 b	5.57 ± 0.47 c	5.18 ± 0.40 c
3	5.01	Furfural	2.50 ± 0.11 a	2.52 ± 0.16 a	2.38 ± 0.13 ab	2.29 ± 0.05 ab	2.11 ± 0.06 b
4	6.61	Methyl hexanoate	5.41 ± 0.39 a	4.56 ± 0.14 b	3.53 ± 0.09 c	2.55 ± 0.20 d	2.01 ± 0.20 d
5	6.78	*α*-Pinene	ND d	2.21 ± 0.01 c	3.19 ± 0.12 c	4.32 ± 0.36 b	5.77 ± 0.76 a
6	7.76	*β*-Myrcene	ND e	2.06 ± 0.05 d	2.97 ± 0.11 c	3.76 ± 0.16 b	4.96 ± 0.59 a
7	7.89	Ethyl hexanoate	2.76 ± 0.36 a	2.20 ± 0.09 b	2.14 ± 0.08 b	1.90 ± 0.15 b	1.76 ± 0.02 b
8	8.00	*α*-Phellandrene	ND e	65.03 ± 0.88 d	91.81 ± 3.97 c	120.57 ± 9.55 b	153.56 ± 14.38 a
9	8.10	3-Carene	ND e	1.03 ± 0.04 d	1.56 ± 0.10 c	2.14 ± 0.17 b	2.79 ± 0.30 a
10	8.21	*α*-Terpinene	ND e	2.04 ± 0.14 d	2.94 ± 0.12 c	3.66 ± 0.16 b	4.47 ± 0.36 a
11	8.34	*p*-Cymene	ND e	7.19 ± 0.20 d	10.27 ± 0.50 c	13.73 ± 0.99 b	16.98 ± 0.76 a
12	8.41	*d*-Limonene	ND e	4.35 ± 0.20 d	6.08 ± 0.23 c	7.63 ± 0.43 b	9.47 ± 0.91 a
13	8.46	Eucalyptol	ND e	23.12 ± 0.78 d	40.49 ± 1.07 c	51.31 ± 3.48 b	65.67 ± 6.32 a
14	8.91	*γ*-Terpinene	ND e	2.82 ± 0.09 d	3.79 ± 0.11 c	4.72 ± 0.28 b	5.88 ± 0.57 a
15	9.39	Terpinolene	ND e	17.28 ± 0.35 d	24.83 ± 0.82 c	32.48 ± 2.21 b	41.01 ± 3.88 a
16	10.80	4-Carvomenthenol	ND e	0.51 ± 0.03 d	1.00 ± 0.08 c	1.20 ± 0.06 b	1.94 ± 0.04 a
17	10.99	*α*-Terpineol	ND e	0.61 ± 0.02 d	1.17 ± 0.03 c	1.46 ± 0.09 b	2.05 ± 0.21 a
18	11.46	5-Hydroxymethylfurfural	5.42 ± 1.73 b	11.44 ± 2.61 a	12.38 ± 1.62 a	10.07 ± 1.02 a	8.91 ± 0.27 ab
19	14.26	*β*-Caryophyllene	ND d	0.72 ± 0.07 cd	1.18 ± 0.03 bc	1.86 ± 0.07 ab	2.64 ± 0.79 a
20	14.60	(*Z*)- *β*-Farnesene	ND c	0.41 ± 0.02 c	0.69 ± 0.04 bc	1.11 ± 0.05 ab	1.48 ± 0.57 a
21	14.98	*ar*-Curcumene	ND d	5.24 ± 0.17 cd	8.19 ± 0.35 bc	12.30 ± 0.44 ab	17.51 ± 5.26 a
22	15.14	*α*-Zingiberene	ND d	104.98 ± 2.81 c	142.89 ± 3.91 bc	209.75 ± 8.35 ab	273.72 ± 60.92 a
23	15.29	*β*-Bisabolene	ND d	9.93 ± 0.40 c	14.21 ± 0.47 bc	21.32 ± 0.90 ab	28.99 ± 7.55 a
24	15.49	*β*-Sesquiphellandrene	ND d	43.47 ± 1.21 c	60.63 ± 2.27 bc	91.12 ± 3.72 ab	122.31 ± 30.95 a
25	15.59	(*E*)-*γ*-Bisabolene	ND d	0.97 ± 0.09 cd	1.83 ± 0.01 bc	2.74 ± 0.11 ab	3.73 ± 1.00 a
26	16.77	*γ*-Muurolene	ND c	5.55 ± 0.68 b	8.40 ± 0.20 ab	8.79 ± 0.60 a	10.16 ± 2.35 a
27	17.15	*ar*-Turmerone	ND d	27.72 ± 2.27 c	43.88 ± 0.85 b	52.49 ± 1.57 b	64.32 ± 8.06 a
28	17.22	*α*-Turmerone	ND d	79.24 ± 4.74 c	114.15 ± 3.55 bc	138.56 ± 3.39 ab	177.39 ± 32.03 a
29	17.58	*β*-Turmerone	ND d	21.26 ± 1.52 c	32.60 ± 1.19 bc	40.32 ± 1.28 ab	53.11 ± 10.74 a
30	18.07	(6*R*,7*R*)-Bisabolone	ND d	2.63 ± 0.26 c	4.32 ± 0.20 bc	5.19 ± 0.25 ab	6.76 ± 1.58 a

Mean values ± standard deviations followed by different letters within a row indicate significant differences using a Tukey’s HSD test at α = 0.05. RT: retention time; ND: not detected.

**Table 3:** Antioxidant properties of turmeric-fortified pineapple (TFP) juice samples (control: 100% pineapple juice; 5%T: 95% pineapple juice + 5% turmeric juice; 10%T: 90% pineapple juice + 10% turmeric juice; 15%T: 85% pineapple juice + 15% turmeric juice; and 20%T: 80% pineapple juice + 20% turmeric juice).

Samples	Total Antioxidant Capacity (Trolox (μM))	DPPH Assay (%DPPH)
Control	766.13 ± 26.03 b	71.51 ± 2.66 c
5%T	972.38 ± 33.08 a	82.13 ± 2.15 b
10%T	986.94 ± 33.24 a	87.22 ± 2.53 a
15%T	1002.63 ± 28.05 a	88.79 ± 0.71 a
20%T	1032.25 ± 42.35 a	90.80 ± 0.99 a

Mean values ± standard deviations followed by different letters within a column indicate significant differences using a Tukey’s HSD test at α = 0.05.

**Table 4:** Phenolic compounds of turmeric-fortified pineapple (TFP) juice samples (control: 100% pineapple juice; 5%T: 95% pineapple juice + 5% turmeric juice; 10%T: 90% pineapple juice + 10% turmeric juice; 15%T: 85% pineapple juice + 15% turmeric juice; and 20%T: 80% pineapple juice + 20% turmeric juice).

Samples	Demethoxycurcumin (mg/L)	Curcumin (mg/L)
Control	ND e	ND e
5%T	2.38 ± 0.07 d	19.40 ± 0.56 d
10%T	5.63 ± 0.23 c	30.10 ± 0.64 c
15%T	9.97 ± 0.05 b	41.71 ± 0.16 b
20%T	11.85 ± 0.13 a	47.50 ± 0.39 a

Mean values ± standard deviations followed by different letters within a column indicate significant differences using a Tukey’s HSD test at α = 0.05. ND: not detected.

**Table 5:** Pearson correlation coefficients between phenolic compounds and antioxidant capacities of turmeric-fortified pineapple (TFP) juice samples.

	Total Antioxidant Capacity	DPPH Inhibition
Demethoxycurcumin	0.76 *	0.90 *
Curcumin	0.87 *	0.96 *

The * values indicate significance at *p* < 0.001.

**Table 6:** Pearson correlation coefficients between sensory scorings, and phytochemical properties and main volatiles of turmeric-fortified pineapple (TFP) juice samples.

	Flavor	Sweetness	Sourness	Mouthfeel	Aftertaste	Overall Quality
*L*	−0.77	−0.81	0.95 *	−0.88 *	0.95 *	−0.39
*a*	−0.89 *	−0.84	0.98 *	−0.98 **	0.84	−0.66
*b*	−0.72	−0.77	0.93 *	−0.85	0.97 **	−0.30
pH	−0.74	−0.78	0.89 *	−0.84	0.88 *	−0.38
TSS	0.79	0.78	−0.98 **	0.96 **	−0.96 *	0.47
TA	−0.81	−0.79	0.93 *	−0.92 *	0.84	−0.55
Demethoxycurcumin	−0.91 *	−0.87	0.99 **	−0.96 **	0.85	−0.67
Curcumin	−0.84	−0.85	0.98 **	−0.93 *	0.93 *	−0.51
Methyl butyrate	0.83	0.70	−0.60	0.60	−0.17	1.00 **
Methyl 2-methylbutyrate	0.86	0.71	−0.78	0.85	−0.48	0.91 *
Furfural	0.82	0.73	−0.88 *	0.94 *	−0.71	0.70
Methyl hexanoate	0.88 *	0.85	−0.99 **	0.97 **	−0.89 *	0.61
*α*-Pinene	−0.84	−0.84	0.96 **	−0.92 *	0.89 *	−0.51
*β*-Myrcene	−0.81	−0.83	0.96 **	−0.91 *	0.91 *	−0.47
Ethyl hexanoate	0.81	0.86	−0.94 **	0.84	−0.91 *	0.43
*α*-Phellandrene	−0.82	−0.84	0.96 **	−0.91 *	0.91 *	−0.48
3-Carene	−0.84	−0.85	0.97 **	−0.93 *	0.90 *	−0.53
*α*-Terpinene	−0.80	−0.82	0.96 **	−0.90 *	0.94 *	−0.44
*p*-Cymene	−0.83	−0.85	0.97 **	−0.91 *	0.92 *	−0.49
*d*-Limonene	−0.80	−0.83	0.96 **	−0.90 *	0.93 *	−0.44
Eucalyptol	−0.82	−0.82	0.97 **	−0.94 *	0.92 *	−0.50
*γ*-Terpinene	−0.80	−0.83	0.95 *	−0.89 *	0.93 *	−0.43
Terpinolene	−0.82	−0.84	0.97 **	−0.91 *	0.92 *	−0.48
4-Carvomenthenol	−0.80	−0.77	0.93 *	−0.93 *	0.86	−0.52
*α*-Terpineol	−0.82	−0.80	0.96 **	−0.95 *	0.90 *	−0.52
5-Hydroxymethylfurfural	−0.05	−0.22	0.38	−0.27	0.72	0.45
*β*-Caryophyllene	−0.88	−0.85	0.96 *	−0.93 *	0.84	−0.61
(*Z*)-*β*-Farnesene	−0.89 *	−0.87	0.97 *	−0.94 *	0.85	−0.62
*ar*-Curcumene	−0.86	−0.85	0.96 *	−0.93 *	0.85	−0.59
*α*-Zingiberene	−0.86	−0.87	0.97 **	−0.91 *	0.88 *	−0.55
*β*-Bisabolene	−0.87	−0.86	0.96 **	−0.92 *	0.86	−0.57
*β*-Sesquiphellandrene	−0.87	−0.86	0.96 **	−0.92 *	0.87	−0.57
(*E*)-*γ*-Bisabolene	−0.87	−0.84	0.97 **	−0.95 *	0.86	−0.60
*γ*-Muurolene	−0.70	−0.74	0.92 *	−0.87	0.98 **	−0.29
*ar*-Turmerone	−0.79	−0.81	0.96 **	−0.92 *	0.95 *	−0.43
*α*-Turmerone	−0.79	−0.81	0.96 *	−0.90 *	0.93 *	−0.43
*β*-Turmerone	−0.81	−0.82	0.96 **	−0.92 *	0.92 *	−0.47
(6*R*,7*R*)-Bisabolone	−0.80	−0.80	0.96 **	−0.93 *	0.93 *	−0.46

The * and ** values indicate significance at *p* < 0.05 and *p* < 0.01, respectively.

## Data Availability

Data is contained within the article.
